# Various Chemical Strategies to Deceive Ants in Three *Arhopala* Species (Lepidoptera: Lycaenidae) Exploiting *Macaranga* Myrmecophytes

**DOI:** 10.1371/journal.pone.0120652

**Published:** 2015-04-08

**Authors:** Yoko Inui, Usun Shimizu-kaya, Tadahiro Okubo, Eri Yamsaki, Takao Itioka

**Affiliations:** 1 Department of Arts and Sciences, Osaka Kyoiku University, Kashiwara, Osaka, Japan; 2 Graduate School of Human and Environmental Studies, Kyoto University, Sakyo-ku, Kyoto, Japan; 3 Center for Ecological Research, Kyoto University, Otsu, Shiga, Japan; Salford University, UNITED KINGDOM

## Abstract

*Macaranga* myrmecophytes (ant-plants) are generally well protected from herbivore attacks by their symbiotic ants (plant-ants). However, larvae of *Arhopala* (Lepidoptera: Lycaenidae) species survive and develop on specific *Macaranga* ant-plant species without being attacked by the plant-ants of their host species. We hypothesized that *Arhopala* larvae chemically mimic or camouflage themselves with the ants on their host plant so that the larvae are accepted by the plant-ant species of their host. Chemical analyses of cuticular hydrocarbons showed that chemical congruency varied among *Arhopala* species; *A*. *dajagaka* matched well the host plant-ants, *A*. *amphimuta* did not match, and unexpectedly, *A*. *zylda* lacked hydrocarbons. Behaviorally, the larvae and dummies coated with cuticular chemicals of *A*. *dajagaka* were well attended by the plant-ants, especially by those of the host. *A*. *amphimuta* was often attacked by all plant-ants except for the host plant-ants toward the larvae, and those of *A*. *zylda* were ignored by all plant-ants. Our results suggested that conspicuous variations exist in the chemical strategies used by the myrmecophilous butterflies that allow them to avoid ant attack and be accepted by the plant-ant colonies.

## Introduction

The tree genus *Macaranga* (Euphorbiaceae) includes many myrmecophytic (ant-plant) species that are distributed in Southeast Asian tropical rain forests. The ant-plants provide their symbiotic ants (plant-ants) nest sites inside hollow stems, and food in the form of food bodies and extrafloral nectaries. Partnerships between *Macaranga* myrmecophytes and their symbiont plant-ants, mainly *Crematogaster* species, are highly species-specific [1,2]. Although the *Macaranga* myrmecophytes are protected by their plant-ants from herbivore attacks [3,4], some specialist herbivores can still be found feeding on *Macaranga* myrmecophytes, such as caterpillars of the genus *Arhopala* (Lycaenidae). Five *Arhopala* species are known to feed on *Macaranga*, including six myrmecophytic species, and each feeds on one or two closely related congeners [5,6]. Natural populations of larvae of the myrmecophyte-feeding *Arhopala* species are quite small [6], and only a small number of eggs are oviposited [5]; thus, most ant colonies on host *Macaranga* myrmecophyte species are not infested by the larvae in the field. Generally, the *Arhopala* larvae, like many other myrmecophilous lycaenid species, bear dorsal nectary organs (DNOs), pore cupola organs, and tentacle organs (TOs) [6]. These specialized organs produce nectars and other substances that are effective in mitigating plant-ant attacks [7]. However, nectars produced by *Arhopala* larvae may be of little importance as a food item for *Macaranga* plant-ants because the encounter probabilities with the larvae by plant-ants is substantially low [6] and, in addition, naturally occurring honeydews of *Coccus* scales, which obligately cohabit with the plant-ants inside the hollow stems of *Macaranga*, are a much more available food source [8,9]. Furthermore, *Arhopala* larvae impose damage to host plants by chewing the leaves. Thus, *Arhopala* larvae are more likely to be antagonistic intruders rather than mutualistic associates for the plant-ants on *Macaranga* myrmecophytes.

The intensity of mutualism between myrmecophilous lycaenids and ants varies among species, from facultative association to obligate nest parasitism [7]. However, irrespective of the intensity, lycaenid larvae are considered to adapt themselves to be recognized by the ants to some degree as the ants’ nestmates or associates because ants normally react aggressively to non-nestmates. In general, ants use cuticular hydrocarbons that are specific to individual ant colonies as key signals for nestmate recognition [10–13]. Reflecting this chemical basis for nestmate recognition in ants, acquiring a composition of cuticular hydrocarbons that are accepted by the host ants is the most common, and almost essential, biological method for myrmecophiles to survive in the ant society [14,15]. Also in myrmecophilous lycaenids, mimicry (biosynthesis) and/or camouflage (acquisition by contact) of a specific blend of cuticular hydrocarbons of the host ant colony have been reported as follows: larvae of a myrmecophilous lycaenid, *Maculinea rebeli*, mimic and camouflage cuticular hydrocarbons of their host ants [16], and larvae of *Niphanda fusca* closely mimic their host males’ hydrocarbons that induce workers’ care [17]. These lycaenid species are both obligate myrmecophiles and social parasites inside the host nests. Meanwhile, chemical profiles of myrmecophiles do not necessarily match well to those of the host ants [18]. In several ant species, workers can learn a specific blend of hydrocarbons insofar as being provided with nectars [19,20]. The associative learning of chemistry and food rewards can be accomplished with one or a few compounds [21,22]. Workers of these ant species, in which cognitive abilities have reported, are typically foragers, however, the plant-ant workers on a *Macaranga* myrmecophyte almost never leave their host plant because they strongly depend for their food on food bodies provided by their host plant and honeydews provided by the symbiont coccids inhabiting the plant-ants’ nest. The plant-ant workers are less likely to recognize lycaenid larvae as associates that provide nectars rather than intruders that should be attacked. Although the larvae of *Arhopala* species that feed on *Macaranga* myrmecophytes do not enter the ants’ nests (*i*.*e*., host stem domatia), the larvae would constantly encounter plant-ant workers because they usually feed on the younger leaves, which are usually patrolled by several or tens of plant-ant workers [6]. Additionally, wounded leaves of *Macaranga* myrmecophytes emit specific volatiles that induce aggression in the plant-ants [23]. Thus, the *Arhopala* larvae feeding on leaves of *Macaranga* myrmecophytes should suffer ant attacks, unless they were to mimic or camouflage the profiles of cuticular hydrocarbons of the plant-ants on their host plants, as other obligate myrmecophilous lycaenids do.

Although larvae of *Macaranga*-feeding *Arhopala* species can grow by feeding on nonhost *Macaranga* myrmecophytic species under ant-free conditions in a laboratory [24], their natural host plant use is highly species-specific, suggesting that the larvae cannot elude attacks by plant-ants associated with nonhost *Macaranga* species. Considering the prevalence of chemical mechanisms to deceive ants in myrmecophilous lycaenids, the specificity of host plant use in the *Arhopala* species allows us to predict that larvae have developed ant-deceiving chemical mechanisms that are specific to the plant-ant species associated with selective *Macaranga* myrmecophyte species. These chemical mechanisms would be required of larvae even if adult females of the *Arhopala* species select their normal host *Macaranga* myrmecophyte species for oviposition sites because plant-ant attacks would be exerted on the larvae that possess DNO-secreted nectar but are not very profitable for the plant-ants. Because *Arhopala* larvae that feed on *Macaranga* myrmecophyte species are considered infrequent herbivores rather than associates, it would be prudent to have chemical profiles that are acceptable to the plant-ants to exploit the host plants.

We hypothesize that each *Macaranga*-feeding *Arhopala* lycaenid is able to elude attacks by plant-ant species on its host *Macaranga* species, but is unable to elude attacks by other plant-ant species colonizing nonhost *Macaranga* myrmecophyte species, and that the cuticular hydrocarbons of lycaenid larvae play an important role in determining the ants’ behaviors in response to the larvae. To test these hypotheses, we evaluated ant responses to experimentally introduced larvae or larval dummies treated with cuticular crude extract, and compared the compositions of cuticular hydrocarbons of plant-ants on *Macaranga* myrmecophytes and the *Arhopala* larvae feeding on *Macaranga* using gas chromatography.

## Materials and Methods

Research permission was given to the all authors from Forest Department of Sarawak, Malaysia in consideration of the Memorandum of Understanding signed between the Sarawak Forestry Corporation and the Japan Research Consortium for Tropical Forests in Sarawak in November 2005.

### Study insects

We targeted three *Arhopala* species that feed on *Macaranga* myrmecophytes and three *Crematogaster* species that are symbiont plant-ants of different *Macaranga* species. All of the examined insects were collected in dipterocarp forests in Lambir Hills National Park (4°20' N, 113°50' E) and Long Semiyang, upper Baram basin (3°10' N, 115°10' E), Sarawak, Malaysia, from 2009 to 2012. Larvae of the three *Arhopala* species, *A*. *dajagaka*, *A*. *amphimuta*, and *A*. *zylda*, have been known to exclusively feed on *Macaranga rufescens*, *M*. *trachyphylla*, and *M*. *beccariana*, respectively [6]. We inspected trees of the three *Macaranga* species to collect the *Arhopala* larvae and the ants. Although most ant species found on myrmecophytic *Macaranga* have not been described, each of the three *Macaranga* species have been known to harbor one or more specific ant species; *Macaranga rufescens* is associated with *Crematogaster* sp.4, *M*. *trachyphylla* is associated with one or two species of the *C*. *borneensis* group, and *M*. *beccariana* is associated with *C*. *decamera* [25].

### Larvae-introduction experiments

We examined plant-ants’ behavioral responses to larvae of the three *Arhopala* species that were experimentally introduced onto leaves of the three *Macaranga* species, *M*. *rufescens*, *M*. *trachyphylla*, and *M*. *beccariana*. For the introduction, second-, third-, or fourth-instar larvae were collected in the field. Each larva was kept in a plastic container in the laboratory for several days before the introduction. In the container, the larva was fed with leaves of its host plant; the plant-ants were removed from the leaves prior to being placed in the container. For the field studies, trees that were 1.5–2.0 m in height, and had no discernable herbivory damage were randomly chosen for each of the three *Macaranga* species. On the abaxial surface of the second top leaf of each tree, one larva was carefully centered with forceps. We observed plant-ant species behavioral response to the introduced larva for 10 min after the introduced larva was first contacted by a worker of the resident plant-ant species. Plant-ant responses were classified into the following three categories: 1) ignoring: no ants being attacked or attended the larva; 2) attending: ants chiefly attended to the larva without biting and sometimes harvested nectars; and 3) attacking: many ants bit the larva. Each larva and each ant colony (tree) was used for a single trial.

A nominal logistic fit was used to assess the effects of the tested plant-ant species and the introduced larval species on the plant-ant responses. Additionally, for each *Arhopala* species, plant-ant responses were compared among plant-ant species using a nominal logistic fit (JMP software, SAS Institute).

### Chemical analysis of cuticular hydrocarbons

Larvae of the three *Arhopala* species and plant-ant workers of their host plant species were collected in the field from six trees of *M*. *rufescens*, *M*. *trachyphylla*, and *M*. *beccariana*. Cuticular hydrocarbons of the insects were extracted on the collection day. Five plant-ant workers and one larva were collected from each of the 18 trees. Because the bodies of the plant-ant workers are very small (1.5–2.5 mm in body length), we lumped five workers on a plant for an extraction. The *Arhopala* larvae that included third and fourth instars (8–16 mm in body length) were much larger than the plant-ant workers. For each of *A*. *dajagaka* and *A*. *zylda*, four third-instar and two fourth-instar larvae were collected. For *A*. *amphimuta*, three third-instar larvae and three fourth-instar larvae were collected. A pool of five workers was soaked together in *n-*hexane for 5 min; each larva was individually soaked in the same manner. The eluate from the larva and the ants was evaporated at room temperature and then dissolved in 50 μl *n*-hexane, including 100 ng *n*-eicosane (as an internal standard), 2 μl of which was analyzed. Analyses were performed with a GC17A gas chromatograph and a QP5050A mass spectrometer (Shimadzu, Kyoto, Japan) equipped with a DB-1HT capillary column (Agilent J&W; Agilent, Santa Clara, CA, USA). The oven temperature was programmed as follows: initial temperature 80°C for 1 min, a first ramp of 25°C/min up to 250°C, and a second ramp of 10°C/min up to 320°C when the temperature was held constant for 10 min. The injector was kept at 300°C and operated in the splitless mode for 0.75 min. The interface temperature was 300°C. Helium was used as the column carrier gas. Retention times of preliminary analyzed *n*-alkane standards (*n*-C20 to *n*-C38) were used to determine the relative equivalent chain length (ECL) in each sequence of samples. To tentatively identify each hydrocarbon, we inspected full-scan mass spectra of each peak. The relative amount of each hydrocarbon within a sample was calculated by comparing the peak area of the compound with that of the internal standard.

Since only *A*. *dajagaka* larvae showed highly similar blends of hydrocarbons with the plant-ants on the host plants (see Results for the details), we compared the similarity in the relative proportion of hydrocarbons that were obtained from the extracts of both species. A relative proportion of each component was calculated as the ratio of the peak area to total peak area of shared peaks. Analysis of similarities (ANOSIM) was applied based on the relative proportions to compare the similarity between species and colonies (trees). A non-metric multidimensional scaling (nMDS) ordination plot was used to visualize the similarities representing a STRESS value. Statistical analyses were performed using R ver. 2.8.1.

### Ant behavioral assay

We examined plant-ants’ behavioral response to the cuticular eluate of the *Arhopala* larvae at various growth stages using Teflon rods (3 mm in diameter, cut to approximately 5 mm in length). The Teflon rods were rinsed with *n*-hexane before use. Chemical analyses of cuticular eluate of the *Arhopala* larvae revealed that fourth-instar larvae had higher concentrations of hydrocarbons compared to third-instar larvae (S1 Table), suggesting that the levels of hydrocarbons increase with larval body size. Therefore, *n*-hexane cuticular eluate of a second-, third-, and fourth-instar larva was applied to one, two, and three Teflon rod(s), respectively, so as to apply roughly same amount of hydrocarbons to each Teflon rod.

For the assay, we collected 30–60 plant-ant workers from each of the 14–18 *Macaranga* trees, and then placed each group of ants in a plastic container (60 mm diameter, 30 mm depth) containing a moistened plaster bottom for at least 2 h before the assay. A piece of stem of the host plant, halved lengthwise, was placed in the plastic container to allow the ants to acclimate. Teflon rods coated with cuticular eluate were placed near the stem piece to begin the trial of each assay. The first 10 antennal contacts to a Teflon rod by the ants were observed and ant behavior after contact was classified into the following categories: 1) ignoring, 2) intensely antennating, and 3) attacking (threatening and biting). When an ant repeatedly contacted a Teflon rod within seconds, only the behavior following the first contact was recorded. A Teflon rod with only *n-*hexane was used as an experimental control. Each Teflon rod was used for one trial. Each ant colony was used for a maximum of three trials of different types of offered chemicals (either of the three larval species and the control) within 2 days.

A multinomial logit model (JMP software, SAS Institute) was fitted with the type of applied chemicals and the plant-ant species as explanatory nominal variables and the ant behavior as the response variable.

## Results

### Response of ants to experimentally introduced *Arhopala* larvae in the field

In the larvae introduction experiments, the plant-ant responses toward the introduced *Arhopala* larvae differed significantly among the three *Arhopala* species (nominal logistic fit, likelihood ratio [LR] χ^2^ = 80.15, *p* < 0.0001), whereas responses did not differ among the plant-ant species (LR χ^2^ = 1.68, *p* = 0.79). The *Arhopala* species × plant-ant species interaction was marginally non-significant (LR χ^2^ = 14.64, *p* = 0.067).

**Fig 1:**
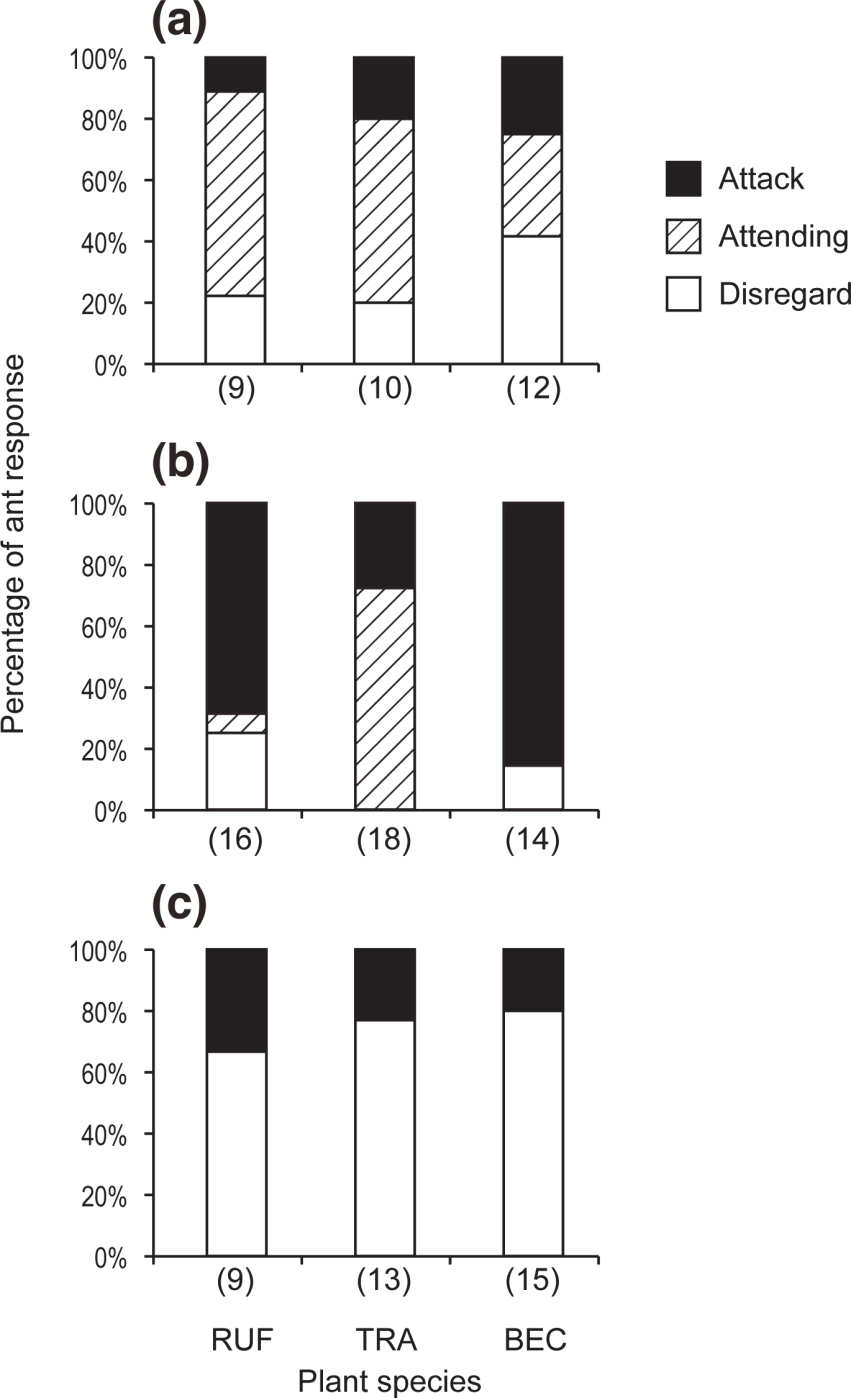
Ant responses to introduced *Arhopala* larvae. Ant responses to larvae of *Arhopala dajagaka* (a), *A*. *amphimuta* (b), and *A*. *zylda* (c) on trees of three *Macaranga* species: *M*. *rufescens* (RUF), *M*. *trachyphylla* (TRA), and *M*. *beccariana* (BEC) are presented. Solid, hatched, and opened columns indicate the percentages of attack, antennation, and ignoring behaviors, respectively. Numbers of tested trees (equal to the numbers of tested ant colonies) are represented in parentheses at the foot of the columns.

*A*. *dajagaka* larvae were often attended, not only by plant-ants on their host plant species, *M*. *rufescens*, but also by other plant-ant species on the nonhost plant species, *M*. *trachyphylla* and *M*. *beccariana* (Fig. 1A). Although the frequency of the three types of ant responses did not significantly differ between the plant-ant species (nominal logistic fit, LR χ^2^ = 2.99, *p* = 0.56), the frequency of ant attacks toward *A*. *dajagaka* larvae was lowest on the host species. The introduced larvae of *A*. *amphimuta* were frequently attacked by plant-ants on the nonhost plant species (*M*. *rufescens* and *M*. *beccariana*) and the frequency of attacks was remarkably lower on their host plant species (*M*. *trachyphylla*; Fig. 1B). Plant-ants on *M*. *trachyphylla* primarily showed attending behavior toward *A*. *amphimuta* larvae (Fig. 1B). The plant-ant responses toward *A*. *amphimuta* differed significantly among the plant-ant species (nominal logistic fit, LR χ^2^ = 32.00, *p* < 0.0001). *A*. *zylda* larvae were chiefly ignored by the plant-ants on all of the host plant species (Fig. 1C), resulting no significant difference in the ant responses among the plant-ant species (nominal logistic fit, LR χ^2^ = 0.54, *p* < 0.76).

*Arhopala dajagaka* larvae were observed to evert the pair of TOs when they were attacked, and the behaviors of the ants were converted to attending within a few minutes. These behavioral interactions were not observed for the other two *Arhopala* species.

### Cuticular hydrocarbons of *Arhopala* larvae and plant-ants

In total, 46 hydrocarbons peaks were obtained from the cuticular extracts of the three *Arhopala* species and the plant-ants from their host plants, including 17 normal alkanes (C23–C39), 23 alkenes, and 13 monomethylalkanes, with a methyl branch on an 11-, 13-, or 15-numbered carbon (Table 1). In all three *Arhopala* species, the total relative amounts of hydrocarbons of fourth-instar larvae were larger than those of third-instar larvae, 1.4, 3.1, and 7.5 times larger in *A*. *dajagaka*, *A*. *amphimuta*, and *A*. *zylda*, respectively, although the differences between instars were not significant (S1 Table). There was no clear qualitative difference in composition of hydrocarbons between the instars.

**Table 1:** Summary of cuticular hydrocabons of plant-ants and *Arhopala* larvae on three *Macaranga* species.

Abbreviation	Compound	ECL	*M*. *rufuscens*		*M*. *trachyphylla*		*M*. *beccariana*	
			ants	*djg*	ants	*amp*	ants	*zyl*
nC23	Tricosane	23	**	*	**			
nC24	Tetracosane	24	*	*	*			
x, y-C25:2	x, y-Pentacosadiene	24.47	**					
x-C25:1	x-Pentacosene	24.71	**	**				
x-C25:1	x-Pentacosene	24.85	**					
nC25	Pentacosane	25	***	***	***	*	**	
11-MeC25	11-Methylpentacosane	25.35	*	*				
x-C26:1	x-Hexacosene	25.88	**					
nC26	Hexacosane	26	**	***		*		
x-C27:1	x-Heptacosene	26.63	***					
x-C27:1	x-Heptacosene	26.71	*	***			**	
x-C27:1	x-Heptacosene	26.89	**					
nC27	Heptacosane	27	***	***	***	**	***	*
11-, 13-MeC27	11-Methylheptacosane and 13-Methylheptacosane	27.33	**	**				
nC28	Octacosane	28	***	***		*	*	
x-C29:1	x-Nonacosene	28.69	***	***				
x-C29:1	x-Nonacosene	28.75	**	**			***	*
x-C29:1	x-Nonacosene	28.83				*		
nC29	Nonacosane	29	***	***	***	**	***	*
11-, 13-, 15-MeC29	11-Methylnonacosane,13-Methylnonacosane and15-Methylnonacosane	29.33	**	**			*	*
x-C30:1	x-Triacontene	29.7	***	*				
x-C30:1	x-Triacontene	29.8					**	*
nC30	Triacontane	30	**	***	***	*		
x,y-C31:2	x, y-Hentriacontadiene	30.46	*	**				
x-C31:1	x-Hentriacontene	30.68	***	***	***			
x-C31:1	x-Hentriacontene	30.77	***	***			***	*
nC31	Hentriacontane	31	***	***	***	**	*	
11-, 13-, 15-MeC31	11-Methylhentriacontane,13-Methylhentriacontane and 15-Methyhentriacontane	31.33	**	**	***	*	*	
x-C32:1	x-Dotriacontene	31.64			***			
nC32	Dotriacontane	32		***				*
x-C33:1	x-Tritriacontene	32.68			***			
nC33	Tritriacontane	33		***		**		
11-, 13-MeC33	11-Methyltritriacontane and 13-Methyltritriacontane	33.34			***			
x-C34:1	x-Tetratriacontene	33.63			*			
nC34	Tetratriacontane	34				*		
x-C35:1	x-Pentatriacontene	34.64			**	*		
x-C35:1	x-Pentatriacontene	34.75				*		
x-C35:1	x-Pentatriacontene	34.85				*		
nC35	Pentatriacontane	35		***		***		
11-, 13-MeC35	11-Methylpentatriacontane and 13-Methylpentatriacontane	35.23			**	*		
nC36	Hexatriacontane	36		***		***		
x-C37:1	x-Heptatriacontene	36.67				*		
x-C37:1	x-Heptatriacontene	36.75				*		
nC37	Heptatriacontene	37		***		***		
nC38	Octatriacontene	38		***		***		
nC39	Nonatriacontane	39		***		***		

Species names of *Arhopala* are represented in three-letter abbreviations as follows; *djg*: *A*. *dajagaka*; *amp*: *A*. *amphimuta*; *zyl*: *A*. *zylda*. Asterisks indicate the frequency of peaks obtained among six samples analyzed

***: obtained from all samples

**: from 4–5 samples

*: from 1–3 samples.

**Fig 2:**
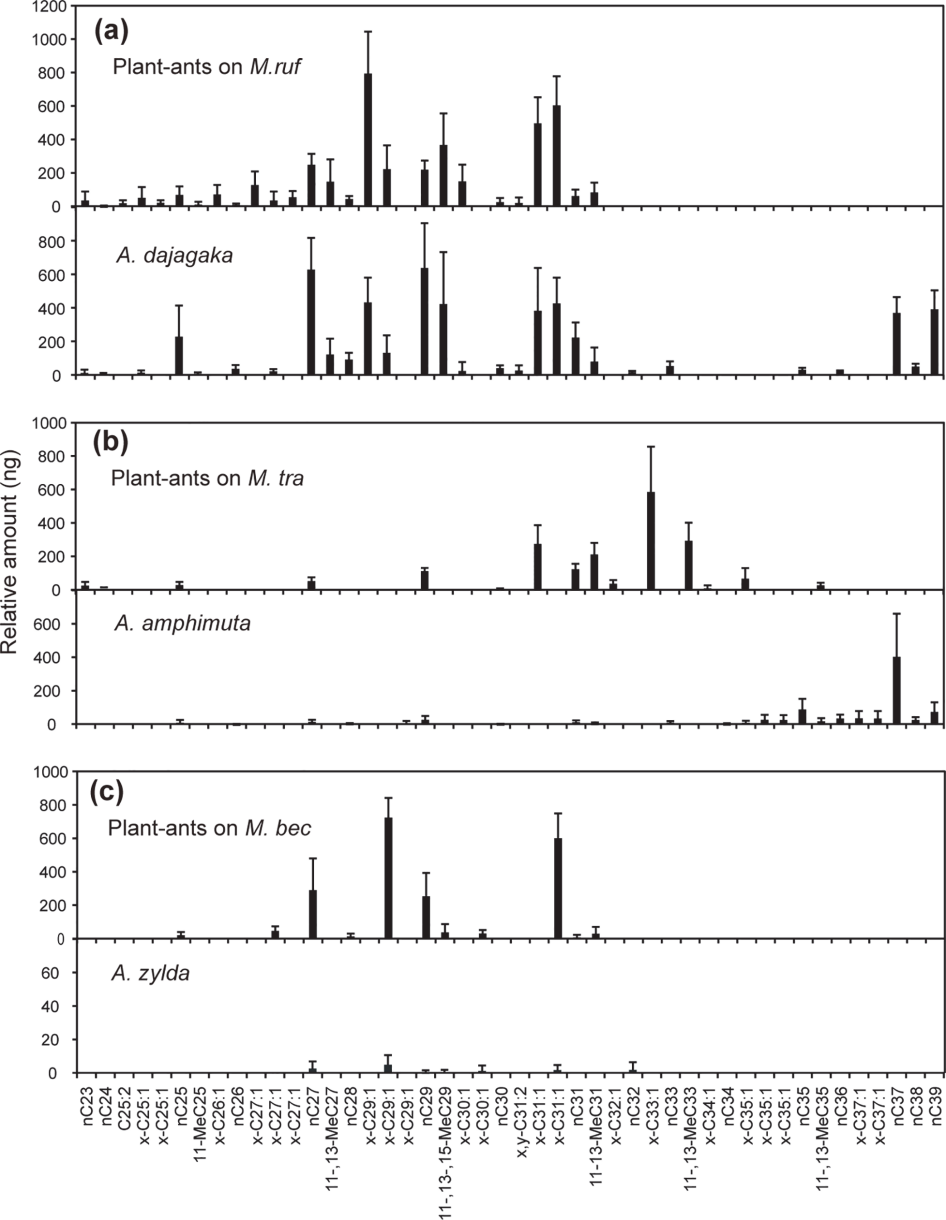
Composition of cuticular hydrocarbons of host ants and *Arhopala* larvae. Columns indicate the mean relative amount of each hydrocarbon per individual of *Arhopala* larvae or per five workers of host plant-ants on (a) *Macaranga rufescens*, (b) *M*. *trachyphylla*, and (c) *M*. *beccariana*. Bars indicate standard deviations. Hydrocarbons are represented by abbreviations as shown in Table 1. For only *A*. *zylda* ((c), lower graph), the vertical axis is shown at an increased scale (x10) compared to the other panels.

**Fig 3:**
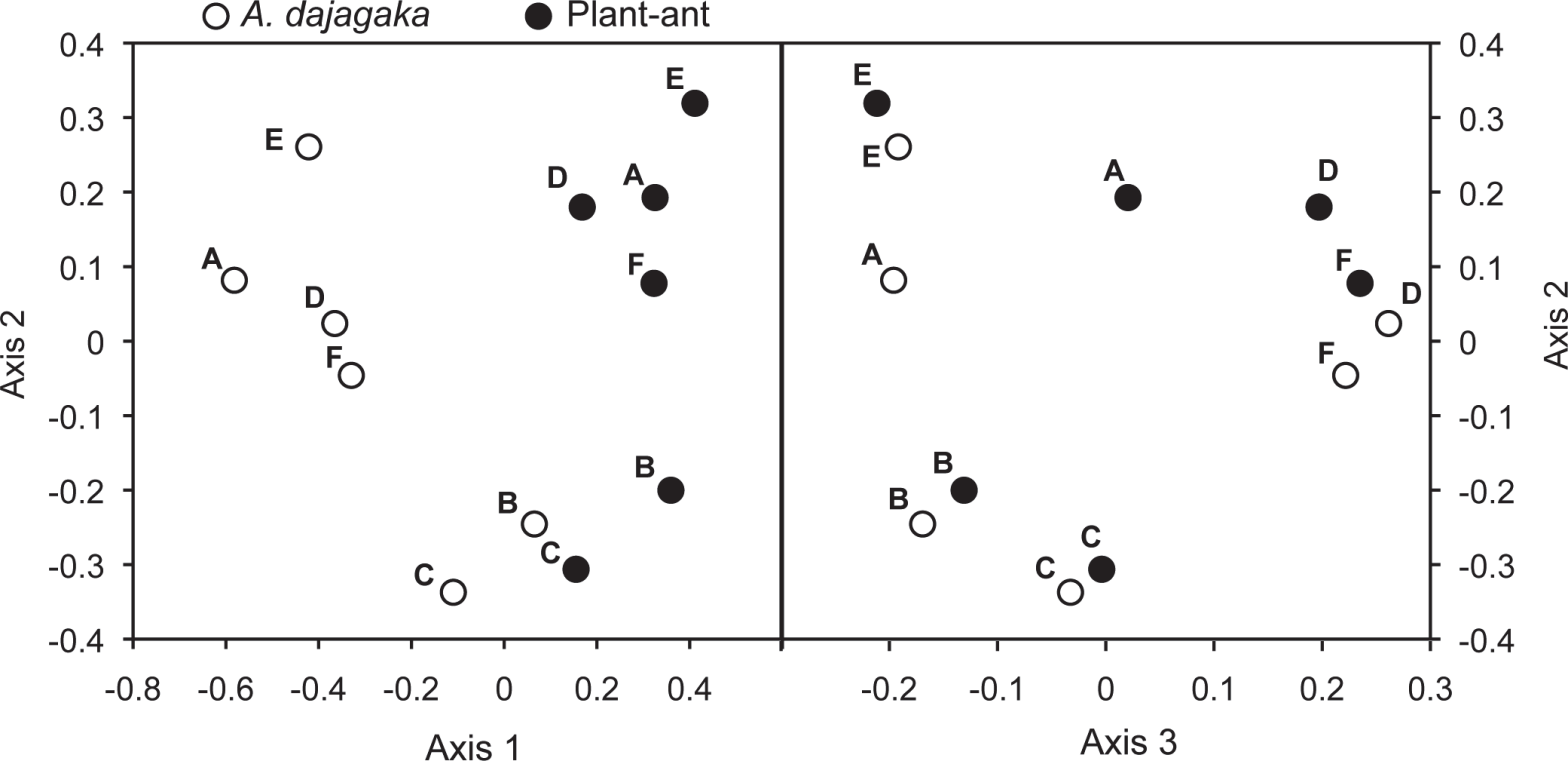
nMDS ordination based on the proportions of shared hydrocarbons of *Arhopala dajagaka* larvae and the host ants collected from six trees of *Macaranga rufescens*. Open and closed circles indicate *A*. *dajagaka* larvae and ant workers, respectively. Alphabetical letters in the facet of axis 2 and 3 indicate the colony identification; same letters indicate the larvae or the ants collected from the same *Macaranga* tree. The MDS stress value was 0.031.

Among the three *Arhopala* species, only *A*. *dajagaka* larvae showed a similar composition of hydrocarbons to the plant-ants on its host myrmecophyte, *M*. *rufescens* (Fig. 2). From *A*. *dajagaka* larvae and the plant-ants on *M*. *rufescens*, 26 and 28 peaks, respectively, were obtained, and 21 components were shared by both species (Fig. 2A, Table 1). On average, the 21 shared components accounted for 81.1% of the total amount of hydrocarbons of *A*. *dajagaka*. The ANOSIM tests based on the relative proportion of the 21 components showed that the dissimilarity between the plant-ants and *A*. *dajagaka* larvae was significant (*R*_ANOSIM_ = 0.72, *P* = 0.004), whereas that among plant-ant colonies was not (*R*_ANOSIM_ = 0.078, *P* = 0.36). However, in the nMDS ordination, the larva and plant-ants of the colony on the same tree were generally clustered tightly along axis 3 (Fig. 3).

From *A*. *amphimuta* larvae and the plant-ants on the host myrmecophyte, *M*. *trachyphylla*, 22 and 15 peaks were obtained, respectively (Fig. 2). *A*. *amphimuta* larvae shared only eight components with the plant-ants, accounting for 10.0% of the total amount of the hydrocarbons. Some compounds with a chain length longer than 36 carbons were obtained from *A*. *amphimuta* larvae, accounting for an average of 71.3% of the total amount of the hydrocarbons that were shared with neither plant-ant species.

From *A*. *zylda* larvae and the plant-ants on the host myrmecophyte, *M*. *beccariana*, seven and 11 hydrocarbons, respectively, were obtained (Fig. 2). Six of the seven components were shared with the plant-ants on different trees; however, the amount of all seven components was minuscule (Fig. 2C). In addition, no component was obtained from more than four larvae among the six larvae used for the chemical analyses (Table 1).

### Response of ants to cuticular extract of *Arhopala* larvae

**Fig 4:**
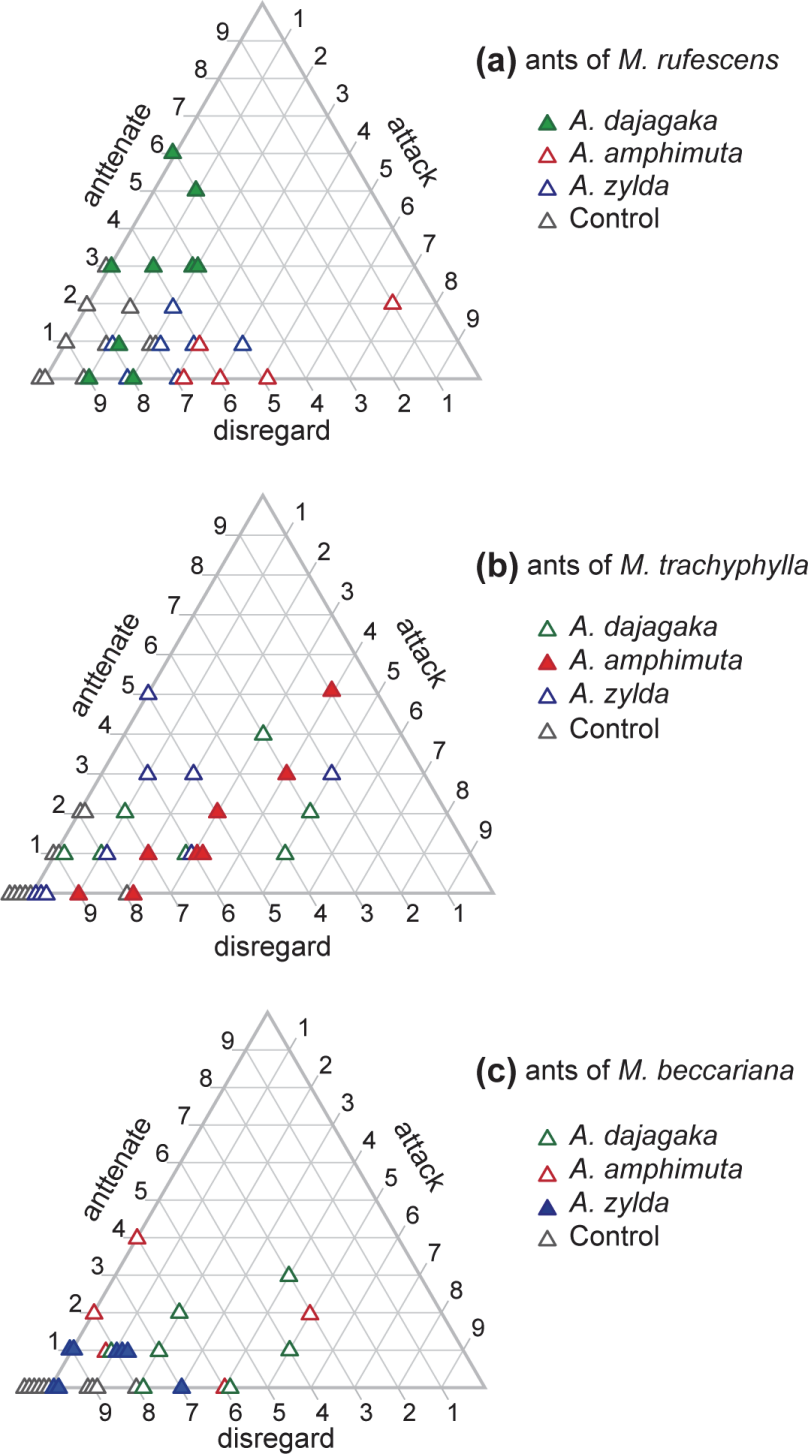
Ant responses to Teflon rods applied with cuticular extract of *Arhopala* larvae. Responses of the first 10 workers toward Teflon rods applied with cuticular crude extract of (a) *Arhopala dajagaka*, (b) *A*. *amphimuta*, and (c) *A*. *zylda*, and (d) *n*-hexane (control) in each trial are plotted. Green, red and blue symbols indicate plant-ant species on *Macaranga rufescens*, *M*. *trachyphylla*, and *M*. *beccariana*, respectively. Overlapping symbols are spread laterally. Filled and open symbols indicate trials for combinations of plant-ant species and *Arhopala* species on the same host plant species and those on the different host plant species, respectively.

In the ant behavioral assays, the plant-ant responses toward the Teflon rod differed significantly among the three plant-ant species (multinomial logit model fit, likelihood-ratio LR χ^2^ = 14.77, *p* = 0.0052) and also among the four types of applied chemicals, *i*.*e*. cuticular extract of larvae of the three *Arhopala* species and n-hexane only (control) (LR χ2 = 97.26, *p* < 0.0001). The plant-ant species × chemical type interaction was significant (LR χ^2^ = 42.79, *p* < 0.001). The workers of the three ant species chiefly ignored the control rods (82%, 92%, and 95% of them on *M*. *rufescens*, *M*. *trachyphylla*, and *M*. *beccariana*, respectively) and rarely attacked them (7%, 4%, and 5% of them on *M*. *rufescens*, *M*. *trachyphylla*, and *M*. *beccariana*, respectively) (S2 Table). Plant-ants of the host species (*M*. *rufescens*) antennated rods applied with cuticular extract from *A*. *dajagaka* larvae, whereas those of the nonhost species (*M*. *trachyphylla* and *M*. *beccariana*) frequently exhibited attacking behavior (Fig. 4A, S2 Table). Toward the rods with cuticular extracts from *A*. *amphimuta* larvae, both plant-ant species of the host (*M*. *trachyphylla*) and nonhost (*M*. *rufescens* and *M*. *beccariana*) myrmecophyte species showed more frequent aggression toward rods coated with cuticular extracts from *A*. *amphimuta* larvae compared with control rods (Fig. 4B, S2 Table). Toward the rods with cuticular extracts from *A*. *zylda* larvae, behavioral responses of plant-ants of the host species (*M*. *beccariana*) toward rods coated with cuticular extracts of *A*. *zylda* larvae were similar to those toward the control rods. Most workers (87.8%) ignored and fewer workers (6.7%) attacked the rods (S2 Table). In contrast, in the plant-ants on the nonhost myrmecophytes of *A*. *zylda* (*M*. *rufescens* and *M*. *trachyphylla*), the frequency of attack toward the rods with cuticular extracts from *A*. *zylda* larvae largely increased (22.5% and 13.3% of ants on *M*. *rufescens* and *M*. *trachyphylla*, respectively) compared with that toward the control rods (Fig. 4C, S2 Table).

## Discussion

The field introduction experiments showed that the responses of the plant-ants to the *Arhopala* larvae varied considerably among *Arhopala* species. However, in all three species, attacks by plant-ants associated with their normal host *Macaranga* species were infrequent.

Larvae of *A*. *amphimuta* were much less frequently attacked and much more frequently attended to by the plant-ants on their host species, *M*. *trachyphylla*, than by those on the two nonhost species. Larvae of *A*. *dajagaka* were also more frequently attended to and less frequently attacked by the plant-ants on their host species, *M*. *rufescens*, although the differences in ant responses were not significant among the plant-ant species. These results suggest that some of the mechanisms used by the two *Arhopala* larvae were used to evade attacks from plant-ants on *Macaranga* myrmecophytes by providing certain secretions and are only effective for the plant-ants on the specific host *Macaranga* myrmecophyte species. The non-significant difference in ant response to *A*. *dajagaka* among the plant-ant species is thought to be partly due to the result that *A*. *dajagaka* larvae seemed to be more frequently accepted by the plant-ants on the nonhost myrmecophytes. However, *A*. *dajagaka* larvae were presumed to be eventually removed by the plant-ants based on observations in preliminary studies where introduced *A*. *dajagaka* larvae to the nonhost myrmecophytes that were not attacked by the plant-ants just after the introduction disappeared before completing their larval periods (T. Okubo and U. Shimizu-kaya, personal observations). This was also observed with all of the *A*. *amphimuta* larvae after introduction to the nonhost myrmecophytes. Although larvae of both *A*. *dajagaka* and *A*. *amphimuta* are suggested to be recognized as intruders by the plant-ants on nonhost *Macaranga*, only *A*. *dajagaka* larvae everted a pair of TOs as if they might tame the attacking ants. Given that only *A*. *dajagaka* larvae were notably frequently attended to by plant-ants on non-host myrmecophytes, TO eversion of *A*. *dajagaka* larvae may have attracted plant-ant workers. However, the exact function and the mechanism of TOs with respect to the maintenance of relationships of ants with lycaenid larvae are still largely unclear, although several studies have suggested the function is to attract [26,27], alert, and/or alarm the ants [28,29]. In addition to TO eversion, *A*. *dajagaka* produce larger droplets of nectar from their DNO more frequently than did *A*. *amphimuta* [6]. The abundant nectar may reward the plant-ants for attending rather than attacking *A*. *dajagaka* larvae, at least in the short term.

In contrast to larvae of the two above-mentioned *Arhopala* species, *A*. *zylda* larvae were chiefly ignored and not so frequently attacked by plant-ants on the host species (*M*. *beccariana*) and on the two nonhost *Macaranga* species; the frequency of ignoring and that of attack did not differ among the plant-ant species. This similarity in the frequency of ignoring and attacking responses among the plant-ants and the generally low frequency of attacks for *A*. *zylda* are presumed to be associated with its myrmecoxenous traits [30]. The results appear to suggest that *A*. *zylda* larvae are unrecognizable, *i*.*e*., myrmecoxenous, even for the plant-ants on the nonhost myrmecophytes. However, the means of eluding plant-ants through myrmecoxeny in *A*. *zylda* larvae would be effective only against plant-ants on their host myrmecophyte species. Five of eight larvae that were introduced to the nonhost myrmecophyte species appeared to "safely" settle themselves without being attacked, and then disappeared before reaching the pupal stage (T. Okubo, personal observation of eight larvae).

Chemical analyses showed that *A*. *dajagaka* larvae had cuticular hydrocarbons that were of similar composition and proportion to the plant-ants on the host species (*M*. *rufescens*). In addition, the cuticular chemicals increased antennation but not attack by the host plant-ants in the behavioral assays. These results suggest that the plant-ants on *M*. *rufescens* accept and attend the *A*. *dajagaka* larvae by recognizing the familiar composition of cuticular hydrocarbons. The cuticular chemicals of *A*. *dajagaka* increased attacks by nonhost plant-ants, suggesting that the ants on *M*. *trachyphylla* and *M*. *beccariana* can chemically recognize *A*. *dajagaka* larvae as intruders. However, the larvae can provide abundant nectar from the DNO so that the larvae themselves were accepted and attended rather than being attacked by the nonhost plant-ants. The TO eversion observed in *A*. *dajagaka* may also play a certain role in inducing the plant-ants on the nonhost myrmecophyte species to attend, although the function of the TOs is still unknown. In the nMDS ordination, the proportions of *A*. *dajagaka* larvae were mapped closer to those of the plant-ants of their own host trees of *M*. *rufescens*. The larvae of an obligate myrmecophilous lycaenid, *Maculinea rebeli* mimic host cuticular hydrocarbons, although composition similarity with that of the host is relatively low; much higher similarity is acquired after colonization [16]. The chemical congruency with ants of *A*. *dajagaka* larvae may also be affected by the hydrocarbons of plant-ants on the trees on which the larvae colonize; *A*. *dajagaka* larvae may be capable of synthesizing or acquiring a more precise blend of hydrocarbons to the plant-ants on their own host trees that allow them safer development.

Results showed that larvae of *A*. *amphimuta* share few common hydrocarbons with the plant-ants on their host species (*M*. *trachyphylla*), suggesting that the larvae do not chemically camouflage themselves as or mimic the plant-ants of the host species. Both plant-ants on the host and nonhost *Macaranga* intended to attack the cuticular chemicals of *A*. *amphimuta*. The plant-ants appear to be capable of recognizing the strange chemical composition of *A*. *amphimuta* as intruders. Despite the incongruence and increased attacks to the cuticular chemicals, *A*. *amphimuta* larvae were accepted and attended by the host plant-ants on *M*. *trachyphylla* in the field introduction experiments. Mechanisms other than chemical mimicry (or camouflage) to evade the ant attacks on host plants likely exist for *A*. *amphimuta*; the myrmecophilous organs such as TOs and DNO are possible mechanisms. Although we have no evidence at present to explain the acceptance of *A*. *amphimuta* larvae by the plant-ants of the host *Macaranga*, a relatively small droplet of nectar from the DNO may be effective to some degree in appeasing the plant-ants. Otherwise, the plant-ants would immediately attack the larvae to remove them from the host trees.

Far fewer hydrocarbons were detected from the larvae of the myrmecoxenous species *A*. *zylda* compared with those detected from the larvae of *A*. *amphimuta* and *A*. *dajagaka* in the chemical analyses, and all of them were in trace amount and obtained from no more than a few individuals. These hydrocarbons are not likely to be stable components of the cuticular lipids of the larvae, but rather negligible contaminated compounds from the plant-ants or laboratory equipment. Therefore, these larvae appeared to lack cuticular hydrocarbons. Hydrocarbons have been said to be essential and major components of insect cuticular lipids of which the primary role is waterproofing and preventing desiccation [31]. *A*. *amphimuta* larvae that did not show mimicry/camouflage to the host plant-ants had many C35–C39 hydrocarbons as likely essential epidermal lipids. Given that we could inspect only hydrocarbons with a chain length of up to 40 carbons and approximately 44 carbons in the GC-MS and on-column GC analyses, respectively, longer chain hydrocarbons may exist on *A*. *zylda* larvae. However, major hydrocarbons among insects range from C20 to C40 in chain length, and in addition, hydrocarbons ranging from C21 to C37 have been reported as definitive components of cuticular lipids of larvae of other lycaenids including *Maculinea rebeli* [16] and *Niphanda fusca* [17]. Both longer hydrocarbons than C40 or so and smaller hydrocarbons up to C40 have been reported to often sufficiently coexist in several insect taxa, such as ants [32], termites [33], and grasshoppers [34]. Thus, the lack of the hydrocarbons in the larvae of *A*. *zylda* seemed to be a striking anomaly among insects. Previous studies have suggested that some social parasites in ant or wasp nests penetrate the host colonies by reducing cuticular hydrocarbons rather than by matching the nestmate signature of the host, so that they may tend to be "chemically insignificant" to the host workers [35,36]. The larvae of *A*. *zylda* may also be chemically insignificant to the plant-ants because most of the ants ignored the Teflon dummies with the larval extracts in the behavioral assays. Moreover, we previously showed highly specialized feeding habits of *A*. *zylda* larvae, which feed mainly on food bodies produced on the abaxial surface of *M*. *beccariana* leaves [24]. Therefore, when compared to other leaf-chewing caterpillars, *A*. *zylda* larvae could cause much less wounding of plant leaves and subsequent volatile emission to induce ant aggression. Both the presence and feeding behavior of *A*. *zylda* larvae seemed to be chemically insignificant to the plant-ants. The host plant-ants and other two *Arhopala* species had no definite components other than hydrocarbons; however, in all of the *A*. *zylda* larvae analyzed, a few compounds were detected in substantial amounts (Inui, unpublished data). These compounds have been tentatively identified as triterpenoids (Inui, unpublished data). In a few insect groups, polar compounds other than hydrocarbons appear to be the dominant components of cuticular lipid [37]. Terpenoids have also been reported as major cuticular compounds in stingless bees, although in co-occurrence with hydrocarbons [38]. Therefore, the *A*. *zylda* terpenoids, instead of hydrocarbons, may play an essential role in the cuticular coat.

Overall, our results suggest that the chemical method of deceiving the plant-ants of the host myrmecophytes is considerably different among the three *Arhopala* species. Larvae of *A*. *dajagaka* were suggested to precisely match the cuticular hydrocarbons of the plant-ants on their host, *A*. *amphimuta* did not match ant cuticular chemistry and may appease the host plant-ants, and the chemical identity of *A*. *zylda* appeared to be concealed. A recent phylogenetic study showed that *Arhopala* species were relatively recently involved in and diversified on already-established *Macaranga* plant–ant symbioses [39]. The intensity of the established mutualistic relationships with the symbiotic plant-ants in terms of intensities of ant and abiotic defenses were found to vary among *Macaranga* species [4,40,41]. Therefore, each of the *Arhopala* species would have been under pressure to become specialized to overcome the specific anti-herbivore defense barrier including the plant-ants. Recent study showed that the degree of chemical congruence between parasitic lycaenid larvae and their host ants is correlated with the intensity of mutualistic relationships with the ants [42]. The conspicuous variation in the chemical strategies within the genus *Arhopala* may be the result of the variation in defense strategies among *Macaranga* myrmechpytes.

## Supporting Information

S1 Table Summary of the larvae used for chemical analyses. Mean ± SE of total relative amount of detected hydrocarbons are shown.
(DOCX)

S2 Table Summary of responses of 10 plant-ant workers to introduced Teflon rods. Total numbers of ant workers of all trials in each combination are shown in Fig. 4.
(DOCX)
