# Supplementary material for: Various Chemical Strategies to Deceive Ants in Three Arhopala Species (Lepidoptera: Lycaenidae) Exploiting Macaranga Myrmecophytes
Source: PLoS One. 2015 Apr 8;10(4):e0120652. doi: 10.1371/journal.pone.0120652 (PMC4390302; doi:10.1371/journal.pone.0120652)
Supplement: S1 Table — Mean ± SE of total relative amount of detected hydrocarbons are shown. (DOCX) [file pone.0120652.s001.docx]

**S1 Table. Summary of the larvae used for chemical analyses.**

|  | Total Amount (ng) | |  |  |
| --- | --- | --- | --- | --- |
| Species | Third instar | Fourth instar |  | Welch’s *P* |
| *A. amphimuta* | 408.2 ± 50.4  (n = 3) | 1276.5 ± 227.5  (n = 3) |  | 0.056 |
| *A. dajagaka* | 4255.2 ± 290.3  (n = 4) | 6130.1 ± 632.6  (n = 2) |  | 0.16 |
| *A. zylda* | 4.3 ± 3.3  (n = 4) | 32.4 ± 17.9  (n = 2) |  | 0.35 |
