# Supplementary material for: Various Chemical Strategies to Deceive Ants in Three Arhopala Species (Lepidoptera: Lycaenidae) Exploiting Macaranga Myrmecophytes
Source: PLoS One. 2015 Apr 8;10(4):e0120652. doi: 10.1371/journal.pone.0120652 (PMC4390302; doi:10.1371/journal.pone.0120652)
Supplement: S2 Table — Total numbers of ant workers of all trials in each combination are shown in Fig. 4. (DOCX) [file pone.0120652.s002.docx]

**S2 Table. Summary of behavioral assays in which responses of 10 plant-ant workers to introduced Teflon rods applied with cuticular crude extracts of larvae of three *Arhopala* species and control rods (applied with *n*-hexane).**

| Chemicals applied to  Teflon rod | Host species of  plant-ants | Number of  trials | Number of ant workers | | |
| --- | --- | --- | --- | --- | --- |
|  |  |  | Ignore | Antennate | Attack |
| Control | *M. rufescens* | 10 | 82 | 11 | 7 |
|  |  |  | (82.0%) | (11.0%) | (7.0%) |
|  | *M. trachyphylla* | 10 | 92 | 4 | 4 |
|  |  |  | (92.0%) | (4.0%) | (4.0%) |
|  | *M. beccariana* | 10 | 95 | 0 | 5 |
|  |  |  | (95.0%) | (0.0%) | (5.0%) |
| *A. dajagaka* extract | *M. rufescens* | 9 | 56 | 24 | 10 |
|  |  |  | (62.2%) | (26.7%) | (11.1%) |
|  | *M. trachyphylla* | 7 | 40 | 12 | 18 |
|  |  |  | (57.1%) | (17.1%) | (25.7%) |
|  | *M. beccariana* | 7 | 42 | 8 | 20 |
|  |  |  | (60.0%) | (11.4%) | (28.6%) |
| *A. amphimuta* extract | *M. rufescens* | 5 | 25 | 3 | 22 |
|  |  |  | (50.0%) | (6.0%) | (44.0%) |
|  | *M. trachyphylla* | 8 | 45 | 13 | 22 |
|  |  |  | (56.3%) | (16.3%) | (27.5%) |
|  | *M. beccariana* | 5 | 31 | 9 | 10 |
|  |  |  | (62.0%) | (18.0%) | (20.0%) |
| *A. zylda* extract | *M. rufescens* | 8 | 54 | 8 | 18 |
|  |  |  | (67.5%) | (10.0%) | (22.5%) |
|  | *M. trachyphylla* | 9 | 62 | 16 | 12 |
|  |  |  | (68.9%) | (17.8%) | (13.3%) |
|  | *M. beccariana* | 9 | 79 | 5 | 6 |
|  |  |  | (87.8%) | (5.6%) | (6.7%) |
